# 14.2 STUCTURED RISK ASSESSMENT IN PSYCHIATRY

**DOI:** 10.1093/schbul/sby014.055

**Published:** 2018-04-01

**Authors:** Seena Fazel, Achim Wolf, Henrik Larsson, Thomas Fanshawe, Susan Mallett

**Affiliations:** 1 University of Oxford; 2 Karolinska Institutet; 4 School of Population and Health Sciences, University of Birmingham

## Abstract

**Background:**

Current approaches to stratify psychiatric patients into groups based on violence risk are limited by inconsistency, variable accuracy, and unscalability.

**Methods:**

Based on a national cohort of 75 158 Swedish individuals aged 15–65 with a diagnosis of severe mental illness (schizophrenic-spectrum and bipolar disorders) with 574 018 patient episodes, we developed predictive models for violent offending through linkage of population-based registers. First, a derivation model was developed to determine strength of pre-specified criminal history, socio-demographic, and clinical risk factors, and tested it in external validation. We measured discrimination and calibration for prediction of violent offending at 1 year using specified risk cut-offs.

**Results:**

A 16 item model was developed from criminal history, socio-demographic and clinical risk factors, which are mostly routinely collected. In external validation, the model showed good measures of discrimination (c-index 0.89) and calibration. For risk of violent offending at 1 year, using a 5% cut off, sensitivity was 64% and specificity was 94%. Positive and negative predictive values were 11% and 99%, respectively. The model was used to generate a simple web-based risk calculator (OxMIV).

**Discussion:**

We have developed a prediction score in a national cohort of patients with psychosis that can be used as an adjunct to decision making in clinical practice by identifying those who are at low risk of violent offending

